# Inflammatory-associated proteomic predictors of cognitive outcome in subjects with ELVO treated by mechanical thrombectomy

**DOI:** 10.1186/s12883-023-03253-z

**Published:** 2023-06-06

**Authors:** Benton Maglinger, Jordan P. Harp, Jacqueline A. Frank, Chintan Rupareliya, Christopher J. McLouth, Shivani Pahwa, Lila Sheikhi, David Dornbos, Amanda L. Trout, Ann M. Stowe, Justin F. Fraser, Keith R. Pennypacker

**Affiliations:** 1 Department of Neurology, Beth Israel Deaconess Medical Center, Boston, MA USA; 2 Department of Neurology, University of Kentucky, Lexington, KY USA; 3 Center for Advanced Translational Stroke Science, University of Kentucky, Lexington, KY USA; 4 Department of Behavioral Science, University of Kentucky, Lexington, KY USA; 5 Department of Neurosurgery, University of Kentucky, Lexington, KY USA; 6 Department of Radiology, University of Kentucky, Lexington, KY USA; 7 Department of Neuroscience, University of Kentucky, Lexington, KY USA; 8 Department of Neurology and Neuroscience, Center for Advanced Translational Stroke Science, University of Kentucky, Building BBSRB, Office B383, Lexington, KY 40536 USA

**Keywords:** Ischemic stroke, Mechanical thrombectomy, Proteomics, Biomarkers

## Abstract

**Background:**

Emergent Large Vessel Occlusion (ELVO) stroke causes devastating vascular events which can lead to significant cognitive decline and dementia. In the subset of ELVO subjects treated with mechanical thrombectomy (MT) at our institution, we aimed to identify systemic and intracranial proteins predictive of cognitive function at time of discharge and at 90-days. These proteomic biomarkers may serve as prognostic indicators of recovery, as well as potential targets for novel/existing therapeutics to be delivered during the subacute stage of stroke recovery.

**Methods:**

At the University of Kentucky Center for Advanced Translational Stroke Sciences, the BACTRAC tissue registry (clinicaltrials.gov; NCT 03153683) of human biospecimens acquired during ELVO stroke by MT is utilized for research. Clinical data are collected on each enrolled subject who meets inclusion criteria. Blood samples obtained during thrombectomy were sent to Olink Proteomics for proteomic expression values. Montreal Cognitive Assessments (MoCA) were evaluated with categorical variables using ANOVA and t-tests, and continuous variables using Pearson correlations.

**Results:**

There were *n* = 52 subjects with discharge MoCA scores and *n* = 28 subjects with 90-day MoCA scores. Several systemic and intracranial proteins were identified as having significant correlations to discharge MoCA scores as well as 90-day MoCA scores. Highlighted proteins included s-DPP4, CCL11, IGFBP3, DNER, NRP1, MCP1, and COMP.

**Conclusion:**

We set out to identify proteomic predictors and potential therapeutic targets related to cognitive outcomes in ELVO subjects undergoing MT. Here, we identify several proteins which predicted MoCA after MT, which may serve as therapeutic targets to lessen post-stroke cognitive decline.

## Background

Emergent large vessel occlusion (ELVO) stroke is one of the leading causes of dementia and disability [19]. For ELVO candidates, endovascular mechanical thrombectomy (MT) has been shown to improve both neurological and cognitive functions in patients when compared to subjects treated with medical therapy alone [33]. However, despite effective strategies to re-establish blood flow, patients still suffer from significant cognitive effects from the injury [15, 28]. In stroke patients, cognitive disability confers a poorer prognosis regarding functional outcomes as well as increased dependence on caregivers [22, 27, 30]. Vascular contribution to cognitive impairment and dementia (VCID), found in 25–30% of stroke patients, is a particularly devastating long-term outcome [12]. While no disease-modifying treatments exist for VCID, early detection may focus medical attention and allow for increased rehabilitation intensity. Thus, novel biomarkers and therapeutic targets will allow for much-needed advances in prognostics and treatment of this devastating disease.

At the University of Kentucky Center for Advanced Translational Stroke Science, human biospecimens obtained from ELVO stroke subjects treated with MT are utilized for research. This Blood And Clot Thrombectomy Registry And Collaboration (BACTRAC) protocol (clinicaltrials.gov; NCT 03153683) allows for processing of both intracranial (distal to thrombus) and systemic (carotid) arterial blood samples. Systemic samples are utilized as acquiring systemic arterial blood is an easier prognostic step than intracranial; however, intracranial blood samples allow for comparison of systemic protein expression with expression at the site of infarction. Using these data, we have reported inflammatory-associated proteomic responses that are predictive of clinical outcomes, such as functional recovery.

Several biomarkers (total tau, pTau181, Aβ_40_, Aβ_42_, Aβ_42/40_), GFAP, and Nfl) of dementia also have been reported to be associated with VCID [3, 4, 13, 25, 31]. However, because stroke is an acute vascular event not always present in ADRD populations, the addition of other potential biomarkers will strengthen the predictive model for determining stroke patients most likely to incur cognitive impairment. The objective of this study was to utilize the BACTRAC registry to identify proteomic biomarkers predictive of cognitive performance at discharge and 90-days in ELVO subjects treated with MT.

## Methods

### Tissue sample and clinical data acquisition

This study utilizes the BACTRAC tissue registry (clinicaltrials.gov; NCT 03153683) of human biospecimens acquired during ELVO stroke in subjects undergoing MT. This study is approved by the University of Kentucky Institutional Review Board (IRB). Inclusion criteria for this study included all ELVO subjects who were candidates for MT and aged 18 or older. Exclusion criteria for this study included age less than18, subjects who were pregnant, incarcerated subjects, and subjects unable to consent within the IRB-outlined 72-h window. Subjects included in this current study were enrolled between June 21, 2017, and March 1, 2021. Methods of acquiring systemic blood during MT per the BACTRAC protocol has been previously published.8 Briefly, arterial blood proximal to the clot is sampled immediately prior to recanalization. Blood is aliquoted into BD Microtainer tubes with K2E (K2EDTA; Becton, Dickinson and Company) and spun down at 2000 rcf for 15 min, plasma is promptly extracted off the top and flash frozen on dry ice in a Wheaton CryoELITE cryogenic vial (DWK Life Sciences; Millville, New Jersey). Samples are stored at -80 C until batches are sent to Olink Proteomics (Olink Proteomics, Boston, MA) for analysis of plasma protein. OLINK proteomics is a high-throughput, multiplexed protein analysis technology that allows for the simultaneous quantification of hundreds of proteins in a single sample.

While BACTRAC enrollment continues, we limited the dataset to this interval to ensure complete primary outcomes. Clinical data are collected on each subject including demographics, comorbidities, relevant labs, radiographic outcome, thrombectomy outcome, and both functional and cognitive outcome metrics. Specific to this study, Montreal Cognitive Assessment (MoCA) scores were either clinically documented in the full 30-point scale or the abbreviated mini-MoCA, 12-point scale. The Montreal Cognitive Assessment (MoCA) is a widely used screening tool to assess cognitive function in adults. It was developed to detect mild cognitive impairment (MCI) and early dementia. The test measures different cognitive domains, such as attention, memory, language, orientation, visuospatial skills, and executive function. The full MoCA test consists of 30 questions and takes approximately 10–15 min to administer. It assesses a wide range of cognitive functions and is sensitive to mild cognitive impairment. The full MoCA test is typically administered by a trained healthcare professional, such as a physician, nurse, or psychologist. The mini-MoCA is a shorter version of the MoCA test, consisting of only 12 questions, and it takes approximately 5–10 min to administer. The mini-MoCA is a quicker and more convenient screening tool for busy healthcare professionals or for use in settings where time is limited. It focuses on the most critical cognitive domains, such as attention, memory, and executive function. A limitation of this study is inconsistent administration of the MoCA or mini-MoCA at discharge by the medical professionals. To remedy this inconsistency in scoring we converted the mini-MoCA score into a 30-point scale for comparability using the following equation.

$$\mathbf P\mathbf r\mathbf o-\mathbf r\mathbf a\mathbf t\mathbf e\mathbf d\boldsymbol\;\mathbf S\mathbf c\mathbf o\mathbf r\mathbf e=\left(30\times\mathbf m\mathbf i\mathbf n\mathbf i-\mathbf M\mathbf o\mathbf C\mathbf A\boldsymbol\;\mathbf s\mathbf c\mathbf o\mathbf r\mathbf e\right)/12$$

### Specimen processing and proteomic analysis

Methods for biospecimen processing for proteomic analysis has been previously published [8, 16–18, 26]. Plasma samples are sent to Olink Proteomics (Olink Proteomics, Boston, MA) for analysis of 96 cardiometabolic and 96 inflammatory proteins. Olink returns proteomic expression values in a Normalized Protein eXpression (NPX) value, which is in log2 scale to reduce intra- and inter-assay variability when running statistics across sample sets. Presently, Olink has been included in over 11,000 publications (https://www.olink.com/).

### Statistical analysis

When assessing for the presence of comorbidities such as hypertension, hyperlipidemia, and diabetic status, unpaired t-tests were utilized. For categorical variables such as location of thrombus (left, right, basilar), and BMI (normal, overweight, and obese) ANOVA was utilized. For continuous variables such as infarct volume, age of patient, and infarct time, Pearson correlations were utilized. Protein concentrations from individual patient samples were assessed with MoCA score at discharge and 90-days using Pearson correlations. For all analyses, *p* ≤ 0.05 was considered significant. Data analysis was performed in GraphPad Prism Version 9.3.1.

## Results

### Subject demographic and comorbid data

Table 1 demonstrates the demographic, comorbid, and outcome data for the separate cohorts of subjects analyzed in this study. There were *n* = 52 subjects with discharge MoCA scores and *n* = 28 subjects with 90-day MoCA scores.

**Table 1:** .

	**MOCA at Discharge**		**MOCA at 90 Days**	
	**Overall Cohort *n* = 52**	**Proteomic Cohort *n* = 23**	**Overall Cohort *n* = 28**	**Proteomic Cohort *n* = 13**
**Age (median; range)**	62 ± 14.7	59 ± 15.1	60 ± 16.5	59 ± 18.7
**Sex**				
Female	29 (56)	15 (65)	10 (36)	5 (38)
Male	23 (44)	8 (35)	18 (64)	8 (62)
**BMI**				
< 18.5	2 (4)	1 (4)	0 (0)	0 (0)
18.5–24.9	10 (19)	5 (22)	5 (18)	2 (15)
25–29.9	15 (29)	8 (35)	9 (32)	6 (46)
> 30	25 (48)	9 (39)	14 (50)	5 (38)
**Comorbidities**				
Hypertension	42 (81)	18 (78)	23 (82)	11 (85)
Diabetes Mellitus II	14 (27)	2 (9)	6 (21)	2 (15)
Hyperlipidemia	16 (31)	5 (22)	8 (29)	4 (31)
Atrial Fibrillation	20 (38)	10 (36)	9 (39)	5 (38)
Previous Stroke	6 (12)	3 (13)	1 (4)	0 (0)
Smoking Status*				
Never	25 (48)	11 (52)	12 (52)	6 (50)
Currently	17 (33)	8 (38)	8 (35)	5 (42)
Previously (> 6 months)	6 (2)	2 (10)	3 (13)	1 (8)
**NIHSS on Admission**				
Minor Stroke (1–4)	3 (6)	1 (4)	2 (7)	1 (8)
Moderate Stroke (5–15)	32 (63)	17 (74)	15 (54)	7 (54)
Moderate/Severe (16–20)	10 (19)	4 (17)	7 (25)	4 (30)
Severe Stroke (≥ 21)	6 (12)	1 (4)	4 (14)	1 (8)
NIHSS at Discharge*				
Minor Stroke (1–4)	38 (74)	16 (70)	16 (59)	6 (50)
Moderate Stroke (5–15)	12 (24)	6 (26)	10 (37)	5 (42)
Moderate/Severe (16–20)	1 (2)	1 (4)	1 (4)	1 (8)
Severe Stroke (≥ 21)	0 (0)	0 (0)	0 (0)	0 (0)
**TICI Score**				
2A = < 50% Perfusion	0 (0)	0 (0)	0 (0)	0 (0)
2B = > 50% Perfusion	19 (37)	9 (39)	11 (44)	6 (46)
3 = Full Perfusion	32 (63)	14 (61)	14 (56)	7 (54)
**LKN to Thrombectomy**	564 ± 356	657 ± 405	625 ± 402	796 ± 460
**Infarct Volume (mm**^**3**^**)**	22,353 ± 27,183	15,991 ± 18,015	32,808 ± 37,961	27,403 ± 36,709
**Location of Thombus**				
Left ICA	2 (4)	1 (4)	1 (4)	1 (8)
Right ICA	1 (2)	0 (0)	0 (0)	0 (0)
Left MCA	16 (30)	6 (26)	8 (28)	1 (8)
Right MCA	28 (54)	14 (61)	14 (50)	9 (69)
Basilar	5 (10)	2(9)	5 (18)	2 (15)
**Source of Thrombus**				
Cardioembolic	32 (61)	14 (59)	15 (53)	7 (53)
Atherembolic	14 (27)	3 (13)	7 (25)	2 (15)
Intracranial Stenosis	1 (2)	1 (5)	1 (5)	1 (8)
Dissection	1 (2)	1 (5)	1 (5)	1 (8)
Carotid Occlusion	1 (2)	1 (5)	1 (5)	1 (8)
Infection	0 (0)	0 (0)	0(0)	0 (0)
Unknown	3 (6)	3 (13)	2 (7)	1 (8)
CTA Collateral Score*				
**0**	2 (9)	2 (10)	4 (24)	3 (25)
1	15 (68)	14 (70)	10 (58)	7 (58)
2	3 (14)	2 (10)	2 (12)	2 (17)
3	2 (9)	2 (10)	1 (6)	0 (0)
	*missing 1 (*n* = 51)	*missing 2 (*n* = 23)	*missing 5 (*n* = 23)	*missing 1 (*n* = 12)
	**missing 3 (*n* = 49)	**missing 1 (*n* = 24)	**missing 1 (*n* = 27)	
	***missing 7 (*n* = 45)	***missing 3 (*n* = 22)	***missing 3 (*n* = 25)	
	****missing 10 (*n* = 42)		****missing 9 (*n* = 19)	
			*****missing 11 (*n* = 17)	

### MoCA scores and subject characteristics

Discharge MoCA scores were assessed in relation to demographic data, comorbidities, and outcome. When location of thrombus was assessed, subjects with a basilar thrombus were found to have significantly lower discharge MoCA scores when compared to the right-sided thrombus group (*p* = 0.01) indicating greater cognitive burden in basilar subjects. No other thrombus location assessments were significant, including no relationship between left- vs. right-sided locations. There was a positive correlation between low-density lipoprotein (LDL) and MoCA score at discharge (*p* = 0.02; R^2^ = 0.12). There were no significant relationships between discharge MoCA scores and patient age, sex, presence of hypertension, hyperlipidemia, diabetes diagnosis, BMI, A1c, TSH levels at presentation, high-density lipoprotein (HDL), triglyceride level, total cholesterol, previous stroke, infarct time, infarct volume, or whether subject received tPA prior to MT.

90-day MoCA scores were also assessed in relation to demographic data, comorbidities, and outcome. Age was found to have a negative correlation with 90-day MoCA scores, indicating older subjects performed worse on the cognitive examination (*p* = 0.03). When subjects were broken down into respective BMI categories (< 24.9 as normal, 25–29.9 as overweight, and ≥ 30 as obese), ANOVA testing revealed the normal weighted group had significantly lower 90-day MoCA scores when compared to the obese group (*p* = 0.03). There were no significant relationships identified when assessing 90-day MoCA scores and patient sex, A1c, TSH, LDL, HDL, triglycerides, total cholesterol; diagnosis of hyperlipidemia, hypertension, diabetes; infarct time, infarct volume, atrial fibrillation or location/source of thrombus.

### MoCA scores and proteomics

Of the *n* = 52 subjects with a discharge MoCA score, *n* = 23 had proteomic data for analysis. Likewise, of the *n* = 28 subjects with a 90-day MoCA score, 13 had proteomic data for analysis. Table 2 demonstrates the top 6 most significant intracranial and systemic proteins correlated with discharge MoCA scores from *n* = 23 subjects. Again, there were no significant relationships identified when assessing MoCA scores and patient sex, A1c, TSH, LDL, HDL, triglycerides, total cholesterol; diagnosis of hyperlipidemia, hypertension, diabetes; infarct time, infarct volume, atrial fibrillation or location/source of thrombus.

**Table 2:** Top 6 most significant intracranial and systemic proteins related to discharge MoCA by *n* = 23 subjects. Proteins were ranked by smallest *p*-values and largest R^2^ values; all correlations were positive

Proteins Significantly Correlated to Discharge MoCA Scores		
Proteins	*P*-value	R^2^ value
*Intracranial Samples*		
SERPINA7	0.01	0.28
DNER	0.01	0.27
APOM	0.02	0.24
IGFBP3	0.03	0.21
s-DPP4	0.05	0.18
MEGF9	0.05	0.18
*Systemic Samples*		
DNER	0.001	0.42
APOM	0.004	0.33
IGFBP3	0.008	0.29
SCF	0.02	0.22
FAP	0.03	0.20
TGFBI	0.03	0.20

**Fig. 1:**
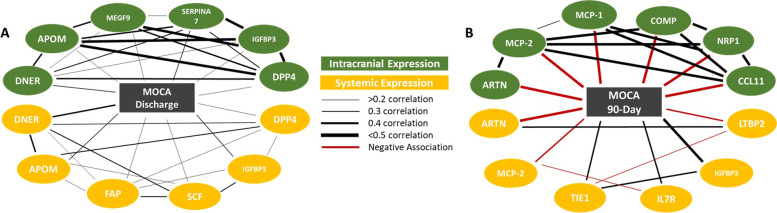
illustrates the inter-protein relatedness among proteins predictive of both MoCA at discharge (**A**) and MoCA at 90-days (**B**). These graphics include both intracranial (green) and systemic (yellow) findings and allow for proteomic comparisons across outcome measures. For example, several of the systemic proteins predictive of MoCA at discharge are also significant in the intracranial blood (DNER, APOM, IGFBP3, s-DPP4), indicating a similar response at the site of infarction compared to blood that could be sampled systemically. These proteomic webs demonstrate network strength (r^2^ value) and aids in the investigation of more complex protein–protein signaling pathways, rather than a singular protein at a specific timepoint

Proteins were ranked by smallest *p*-value and largest R^2^ value. Most significant intracranial proteins correlated to discharge MoCA score (all with positive correlations) include thyroxine-binding globulin (SERPINA 7), delta and notch-like epidermal growth factor-related receptor (DNER), apolipoprotein M (APOM), insulin-like growth factor binding protein-3 (IGFBP3), soluble dipeptidyl peptidase-4 (s-DPP4), and multiple epidermal growth factor-like domains protein 9 (MEGF9). Most significant systemic proteins correlated to discharge MoCA score (all with positive correlations) include DNER, APOM, IGFBP3, stem cell factor (SCF), prolyl endopeptidase FAP (FAP), and transforming growth factor-beta-induced protein ig-h3 (TGFBI) (Fig. 1).

Table 3 demonstrates the top 6 most significant intracranial and systemic proteins related to discharge MoCA scores from *n* = 13 subjects. Proteins were ranked by smallest *p*-value and largest R^2^ value. Most significant intracranial proteins correlated with 90-day MoCA (all were negative correlations) include artemin (ARTN), monocyte chemotactic protein-2 (MCP-2), monocyte chemotactic protein 1 (MCP-1), cartilage oligomeric matrix protein (COMP), neuropilin-1 (NRP1), and eotaxin (CCL11). Most significant systemic proteins negatively correlated with 90-day MoCA scores include ARTN, latent-transforming growth factor beta-binding protein 2 (LTBP2), and MCP-2. Most significant systemic proteins positively correlated with 90-day MoCA scores include insulin-like growth factor binding protein-3 (IGFBP3), tyrosine-protein kinase receptor tie-1 (TIE1), and interleukin-7 receptor subunit alpha (IL7R).

**Table 3:** Top 6 most significant intracranial and systemic proteins related to 90-day MoCA. Proteins were ranked by smallest *p*-values and largest R^2^ values. Asterisks indicate negative correlation while proteins without asterisks indicate positive correlations

Proteins Significantly Correlated to 90-day MoCA Scores		
Proteins	*P*-value	R^2^ value
*Intracranial Samples*		
ARTN*	0.002	0.59
MCP-2*	0.003	0.57
MCP-1*	0.003	0.56
COMP*	0.007	0.46
NRP1*	0.007	0.49
CCL11*	0.01	0.46
*Systemic Samples*		
ARTN*	0.001	0.62
IGFBP3	0.01	0.45
LTBP2*	0.02	0.43
TIE1	0.02	0.43
IL7R	0.02	0.42
MCP-2*	0.02	0.42

## Discussion

Thrombectomy guidelines for ELVO stroke subjects have been generated by trials which measured neurologic/functional outcome, often the modified Rankin Score [34]. We aimed to utilize systemic and intracranial proteomic data on ELVO subjects undergoing MT to identify proteomic biomarkers of cognition which may be both prognostic as well as targets for novel/existing therapies. Systemic blood is reliably accessible for potential prognostics while the analysis of intracranial blood reveals the local ischemic response, which identifies potential therapeutic targets.

We started by investigating patient demographic data for predictors of cognitive performance. When assessing discharge MoCA scores, aside from basilar location of thrombus and LDL levels, there were no significant relationships with demographic/laboratory data nor infarct time. Our finding of a positive correlation between LDL and discharge MoCA scores but not 90-day MoCA scores may be related to atherosclerotic burden at presentation; however, studies have reported few cognitive consequences related to chronic LDL levels [20]. When assessing 90-day MoCA scores, age was found to have a negative correlation, whereas having normal BMI was associated with lower cognitive scores. Both findings are unsurprising as age-related cognitive decline is well-known, and our group has previously reported obese stroke patients are significantly younger (17 years) compared to the normal BMI cohort [17].

Next, we investigated systemic and intracranial proteins found to have significant correlations to discharge MoCA scores as well as to 90-day MoCA scores. Interestingly, several of the proteins we found to have a significant relationship with post-stroke cognitive function have been previously reported to play a role in stroke outcome and cognition/neurodegeneration.

First, we focus on soluble dipeptidyl peptidase 4 (s-DPP4) and C–C motif chemokine 11 (CCL11) as biomarkers and potential therapeutics for post-stroke cognition that have been previously reported on in the context of stroke and in cognition. Soluble DPP-4 (s-DPP4) is well-known in the diabetes literature leading to the development of several inhibitors which help lower blood glucose levels. FDA-approved DPP4 inhibitors typically block the membrane bound form of DPP4, which increases s-DPP4. In our current study, we studied the soluble form of DPP4 and found a positive correlation between intracranial s-DPP4 and discharge MoCA scores indicating higher s-DPP4 was predictive of better cognitive function. DPP4 inhibitors have previously been administered in several rodent models of stroke and have demonstrated efficacy in reducing injury and enhancing functional recovery [5]. Further, these inhibitors have been associated with improvement in cognition in a diabetic rat model and have been suggested as a potential treatment for Alzheimer’s disease [1, 24]. Our findings that increased s-DPP4 (a potential consequence of DPP4 inhibition), was predictive of better cognitive function corroborate prior findings in the stroke and cognition literature. In our study, we also found that intracranial CCL11 was negatively correlated with 90-day MoCA scores. This finding is unsurprising as CCL11 has been shown to be a causative factor in the cognitive decline of aging [35]. CCL11 is a ligand for the chemokine receptor type 3 (CCR3) receptor and, thus, CCR3 has been identified as a potential therapeutic target for Alzheimer’s disease that reduces amyloid beta deposition and tau phosphorylation [35]. Interestingly, DPP4 has been shown to cleave CCL11 and reduce its chemotactic interaction with CCR3 [29]. Taking previous finding into the context of our current study, we postulate s-DPP4 exerts a beneficial effect on cognition after ELVO by cleaving and inactivating chemokines such as CCL11 that impair cognition through the CCR3 receptor pathway. This supports existing literature that DPP4 inhibitors may be useful in combatting cognitive decline and offers a specific human pathology for future application.

Additional proteins which have been shown to be related to neurodegeneration include IGFBP3, DNER, and NRP1. Insulin-like growth factor-binding protein 3 (IGFBP3) is one of six members of a family known to carry IGF-1. In our study, we found that intracranial IGFBP3 was positively correlated to discharge MoCA score and similarly systemic IGFBP3 was positively correlated to both discharge and 90-day MoCA scores indicating higher IGFBP3 were predictive of better cognitive function. A prior study reported that low levels of IGFBP3 were predictive of worse functional outcome at one-year post-stroke based on modified Rankin scores [6]. Our findings align with and add to this study by including cognitive function metrics after stroke. A separate study investigating insulin-like growth factors and cognitive function in the aging male population reported increased IGFBP3 was significantly associated with greater cognitive decline in their studied population [10]. Interestingly, the directionality of our findings are opposite to this study, which may offer a unique relationship between IGFBP3 and cognition in stroke patients specifically. Delta and Notch-like epidermal growth factor-related receptor (DNER) has been shown to activate the NOTCH1 pathway which has been reported to contribute to neurodegeneration and Alzheimer’s pathophysiology [23]. In our study, both systemic and intracranial DNER were positively correlated with discharge MoCA scores but not at the later time point of 90-days, indicating a potential temporal change in the proteomic expression which may influence cognitive function in the sub-acute phase of recovery. Neuropilin-1 (NRP1) has been shown to be upregulated in patients with severe Alzheimer’s disease [14]. One study reported NRP1 to interact with APOE-e4 in cognition as higher levels of NRP1 correlated to cognitive decline in patients with the APOE-e4 gene [21]. NRP1 has been shown to have a role in mitochondrial dysfunction, atherosclerosis, and neurodegeneration as well as in brain microvascular endothelial inflammation and blood–brain-barrier function [2, 32]. Not surprisingly, vascular dysfunction and blood–brain-barrier disruption have both been shown to be directly related to VCID [7]. In our cohort, NRP1 was found to be negatively correlated with 90-day MoCA scores indicating higher levels were associated with worse cognitive function as supported by prior literature.

Other proteins that stood out in our findings include MCP1 and COMP. A previously published meta-analysis reported increased circulating levels of monocyte chemotactic protein 1 (MCP1) was associated with increased long-term risk of stroke and that this protein may serve as a potential therapeutic target [9]. In our study, we add to the existing literature by reporting on MCP1 and cognitive outcome after stroke. Like the meta-analysis, we found that increased MCP1 levels were deleterious; specifically, intracranial MCP1 levels were negatively correlated with 90-day MoCA scores. A previous study reported cartilage oligomeric matrix protein (COMP) to be positively associated with worse plaque burden and plaques that were symptomatic in carotid atherosclerosis [11]. Again, our study adds to the existing literature by reporting on the relationship between COMP levels and cognitive function post-stroke. Like the prior study, we found COMP to be a negative factor; specifically, we report a negative correlation between intracranial COMP levels and 90-day MoCA scores indicating higher COMP levels predicted worse cognitive function at the 90-day time point. As many ELVO strokes are atherosclerotic in etiology, we find the relationship between COMP and MoCA to be of particular interest in future studies.

Several biomarkers related to dementia are also linked to the development or prediction of stroke-induced dementia. Plasma levels of Ab42/40-b and ptau181 and total tau have been reported to be involved in the development of post-stroke cognitive impairment [3, 4, 31]. Both of these proteins are also associated with cerebral microbleeds, which is a risk factor for dementia. 32 Higher plasma NfL has been reported to be predictive of unfavorable functional outcomes after stroke. 33 GFAP has been reported to provide clinical information in differential diagnosis of different types of strokes 10.1007/BF03256432. These biomarker studies use patient data after the stroke and don’t differentiate in the type of ischemic stroke. Additional studies are needed to determine if these biomarkers are present at the time of thrombectomy which is 3–12 h after the last known normal.

An existing limitation of BACTRAC is the geographic location where samples were collected. Our study represents one population of the United States with limitations on diversity, mainly serving Caucasian individuals with homogenous comorbidities. However, a significant portion of our stroke patients are from rural areas of Appalachia, which represents a population with known health disparities. Access to this patient population will allow us to further study an underserved area where novel prognostics and therapeutic interventions would be greatly valued. Proteomic relationships with stroke outcomes in Appalachia will be the focus of subsequent studies conducted by our group. Another limitation of this study is the sample size of subjects with discharge and 90-day MoCA scores. The full MoCA is most appropriate for individuals with at most moderate impairment, as aphasia and other more severe impairments can interfere mask otherwise intact abilities (receptive language, verbal memory) on some items on the test. For such patients we collected a Mini-MoCA better suited to the population. There remains a potential selection effect, as only those with a MoCA or MiniMoCA score were included in the analysis, and thus our findings may be limited to those without profound post-stroke impairment. Data reported here will be validated as BACTRAC enrollment continues and larger analyses are conducted. Another constraint of this study is that it is limited to correlative analyses. For example, some proteins are elevated because of vascular injury and contribute to the injury, however, some proteins are consequentially upregulated as a response/protective/rescue measure. Further, some proteins may have high expression but lower activity or vice versa. However, these correlations still could serve as predictive biomarkers for cognitive performance after stroke. It is also important to emphasize we are studying cognitive decline secondary to ELVO treated by MT, which is a very specific pathophysiology in a very specific cohort of patients. ELVO injury is significantly different from other types of stroke and small vessel disease and the cognitive decline after ELVO is likely different from cognitive decline secondary to dementias of varying etiologies. Lastly, proteins which have been shown to contribute to stroke severity focus on outcome metrics different from cognitive function tests, for example mRS. Here we provide preliminary but novel data on systemic and intracranial protein expression in ELVO subjects treated with MT and how those proteins related to cognitive function at the time of discharge as well as at 90-day follow-up.

## Conclusion

In conclusion, we set out to identify proteomic predictors and potential therapeutic targets related to cognitive outcomes in ELVO subjects undergoing MT. Here, we report several proteins which were found to be predictive of MoCA scores at discharge and at 90-days. Many proteins reported here such as s-DPP4 and CCL11 have been studied in the context of cognition previously, however, our study investigates their role in a specific population after endovascular recanalization. These proteins serve as a springboard for future therapeutic applications to offset stroke-related cognitive decline.

## Data Availability

Data available upon request to the corresponding author, Keith Pennypacker, PhD.

## Abbreviations

ELVO: Emergent Large Vessel Occlusion
MT: Mechanical thrombectomy
BACTRAC: Blood And Clot Thrombectomy Registry and Collaboration
MoCA: Montreal Cognitive Assessment
VCID: Vascular contributions to cognitive impairment and dementia
NPX: Normalized protein expression
BMI: Body mass index
TICI: Thrombolysis in cerebral infarction
NIHSS: National institute of health stroke scale/score
SERPINA 7: Thyroxine-binding globulin
DNER: Delta and notch-like epidermal growth factor-related receptor
APOM: Apolipoprotein M
IGFBP3: Insulin-like growth factor binding protein-3
s-DPP4: Soluble dipeptidyl peptidase-4
MEGF9: Multiple epidermal growth factor-like domains protein 9
SCF: Stem cell factor
FAP: Prolyl endopeptidase
TGFBI: Transforming growth factor-beta-induced protein ig-h3
ARTN: Artemin
MCP-2: Monocyte chemotactic protein-2
COMP: Cartilage oligomeric matrix protein
MCP-1: Monocyte chemotactic protein 1
NRP1: Neuropilin-1
CCL11: Eotaxin
LTBP2: Latent-transforming growth factor beta-binding protein 2
TIE1: Tyrosine-protein kinase receptor tie-1
IL7R: Interleukin-7 receptor subunit alpha
